# Age-related and obstacle height-related differences in movements while stepping over obstacles

**DOI:** 10.1186/s40101-015-0052-8

**Published:** 2015-03-24

**Authors:** Sohee Shin, Shinichi Demura, Tsuneo Watanabe, Tamotsu Yabumoto, Jae-Hyun Lee, Naoki Sakakibara, Toshio Matsuoka

**Affiliations:** Department of Sports Medicine and Sports Science, Gifu University Graduate School of Medicine, 1-1 Yanagido, Gifu City, 501-1194 Japan; Graduate School of Natural Science & Technology, Kanazawa University, Kakuma, Kanazawa, Ishikawa, 920-1164 Japan; Korea University, 145, Anam-ro, Seongbuk-gu, Seoul, South Korea

**Keywords:** Age-related difference, Obstacle height-related difference, Stepping over obstacles

## Abstract

**Background:**

This study aims to examine age-related and obstacle height-related differences in movements while stepping over obstacles.

**Methods:**

The participants included 16 elderly and nine young women. Obstacles that were either 5 or 20 cm high were positioned at the center of a 4-m walking path. The participants were instructed to walk along the path as quickly as possible. The participants’ movements were analyzed using a three-dimensional motion analysis system that recorded their movements as they walked and stepped over the obstacles.

**Results and conclusions:**

Seven joint angles and the distances between the ground and six markers were examined in the initial contact and swing instants of the leading and trailing limbs. In the initial contact instant, the elderly women prepared for stepping with a lower toe height than the young women when stepping over the 20-cm obstacle. Trunk rotation was greater in the young women than in the elderly women. In the swing instant, the elderly women showed greater ankle dorsiflexion and hip adduction angles for the leading limb when stepping over the 20-cm obstacle. They moved the trailing limb with increased ankle dorsiflexion, knee flexion, hip flexion, and foot inversion to ensure that they did not touch the obstacle as they stepped over it. These movement patterns are characteristic of elderly individuals who cannot easily lift their lower limbs because of decreased lower-limb strength.

## Background

The population of individuals aged over 60 years is growing faster than that of individuals in any other age group worldwide, and this population is predicted to balloon from 688 million in 2006 to almost 2 billion by 2050 [1]. Suzuki et al. [2] and Demura et al. [3] examined the conditions that lead to falls in community-dwelling elderly individuals and found that the incidence of falls over a 1-year period was approximately 20% in elderly individuals aged 65 years and over, albeit with some regional differences. On account of the incidence of falls increasing with age, the possibility that elderly individuals would suffer fractures and become bedridden also increases. A decline in physical fitness significantly limits the activities of daily living (ADL) in the elderly and further increases the possibility of falls [4].

Most falls among the elderly occur while walking [5]. Therefore, it is necessary to improve our understanding of the aging-related gait changes. The characteristics of walking movements in the elderly generally include decreased walking speed, shortened stride, long double-support phase, decreased foot elevation during the swing phase, broad-based gait, decreased upper-limb swing, and instability during direction changes [6].

In contrast, the mechanisms underlying falls in the elderly include tripping, slipping, misstepping, and staggering. The differences of these mechanisms necessitate different optimal prevention strategies. According to previous studies [7,8] that examined the causes of falls, most falls occur when elderly individuals trip while walking. Chen et al. [9] reported that falls associated with tripping over obstacles can lead to devastating consequences for elderly individuals. Furthermore, the same study, which examined individuals crossing obstacles, added that older adults showed a more conservative strategy, using a slower crossing speed, shorter step length, and shorter obstacle-heel strike distance while crossing obstacles. Lu et al. [10] reported that older individuals adopted a swing hip flexion strategy to increase leading toe clearance. Compared with younger individuals, older individuals showed a linear increase in their leading toe clearance with increasing obstacle height, and this pattern is achieved by changing fewer joint angular components. This strategy allowed older individuals to maintain stability with minimum effort.

On the other hand, several studies to date have evaluated various effects of stepping over obstacles on the elderly, such as the effect of lower-limb muscle fatigue and age [11,12], clearance of the leading and trailing limbs [13], trailing toe-obstacle distance [14] and walking speed [15], center of gravity (COG) characteristics [16], joint angle [13] and lower-limb kinematics [10], and proprioception when stepping over obstacles.

Most studies that examined elderly individuals stepping over obstacles have focused mostly on time-series data recorded during the entire gait cycle [13,16]; however, few studies have examined specific points in the gait cycle. The phase during which the elderly show these characteristic movements remains uncertain. In this study, these movements were evaluated by analyzing gait at the instance of initial contact or swing instants for the leading and trailing limbs. Furthermore, the researchers compared the movement characteristics of elderly and young individuals stepping over obstacles. The objective is to examine age-related and obstacle height-related differences among individuals who stepped over obstacles.

## Methods

### Participants

The participants were 16 healthy elderly women who could walk independently (mean age, 73.7 ± 4.4 years; mean height, 147.5 ± 4.8 cm; mean weight, 52.1 ± 7.3 kg) and nine young women who served as the control group (19.6 ± 1.4 years, 162.5 ± 5.2 cm, 57.3 ± 6.4 kg). The mean activities of daily living (ADL) score, which was evaluated according to the criteria established by the Ministry of Education, Culture, Sports, Science, and Technology of Japan, was 28.1 ± 3.8. This was similar to the score (28.9 ± 3.9) described in a previous study [17] that examined healthy elderly individuals. In addition, an ADL questionnaire was administered to only the elderly women. The purpose and procedures of this study were explained to all the participants in detail, and written informed consent was obtained prior to their participation in the study. This study was approved by the institutional review board of the Gifu University School of Medicine (reference number, 24-310).

### Obstacle gait

**Figure 1:**
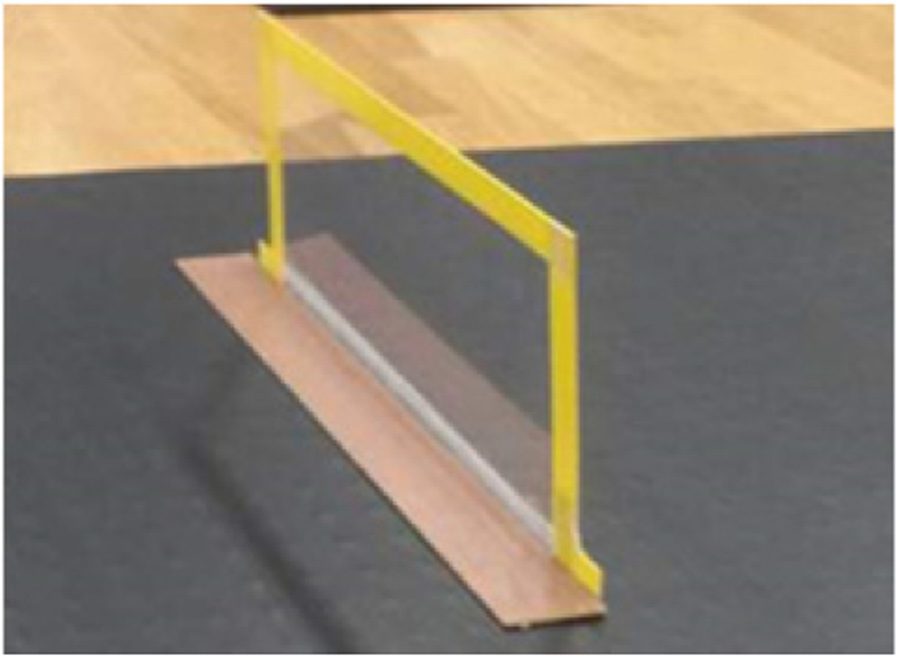
**Obstacle in this study.**

Participants walked for 4 m in the absence of obstacles (0-cm obstacle) and in the presence of obstacles measuring 5 and 20 cm in height. A thin, translucent acrylic plate (length: 45 cm, thickness: 2 mm) was installed to prevent tripping and falling to progress direction even if the participant kicked it. In addition, a clear acrylic plate was used to record markers, regardless of whether they were obscured by the obstacle. In addition, color tape was attached to all sides of every obstacle to indicate its height (Figure 1). The obstacles were positioned at the center of the walking path. Participants were instructed to walk the 4-m distance and step over the obstacles as quickly as possible. The participants’ movements were recorded and measured using a three-dimensional motion analysis system (Kinema Tracer; Kissei Comtec, Nagano, Japan) as they walked and stepped over the obstacles. The motion analysis system recorded the participants’ movements using markers attached to the shoulder, greater trochanter, knee, heel, and toe. The Kinema Tracer includes charge-coupled device (CCD) cameras and a computer for recording and analysis. This system is convenient for three-dimensional motion analysis. IEEE 1394 cables connected the CCD cameras to the computer, which eliminated the need to synchronize the cameras during video recordings. Kinematic parameters were sampled at 60 Hz. The control object was recorded with four cameras for the calibration frame, and the coordinates for the control object were specified using the direct linear transformation method. After taking these measurements, ten markers were digitized using an automatic tracking function but were digitized manually when the automatic tracking failed because the markers were not visible in the camera images.

### Evaluation instants and parameters

**Figure 2:**
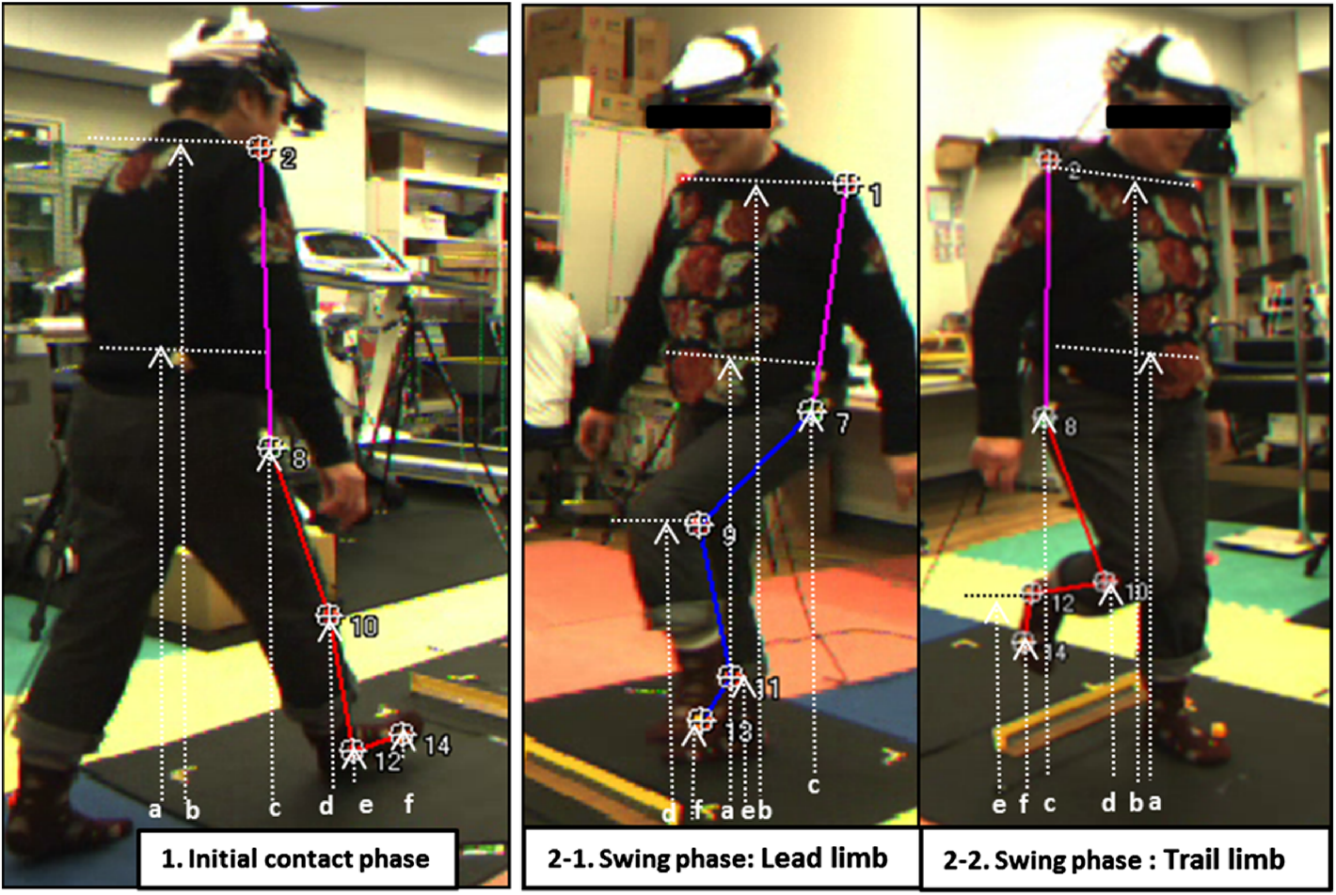
**The distances between the markers and the ground.** During the initial contact (left) and swing phases (middle and right). (a) Center of gravity, (b) shoulder, (c) great trochanter, (d) knee, (e) ankle, (f) toe.

This study examined stepping movements over obstacles by examining the angles of the trunk, hip, knee, and ankle as well as the marker heights from the ground during the initial contact and swing instants for both the leading and trailing limbs. We evaluated and analyzed the below mentioned movement instants (Figure 2).

1-1) Initial contact instant
  The point at which the supporting lower limb first contacted the ground before the leading lower limb stepped over the obstacle.
1-2) Swing instant (a. leading limb, b. trailing limb)
  The points at which the leading or trailing limb reached its maximum height during the swing phase.

The evaluation parameters are described below.

**Figure 3:**
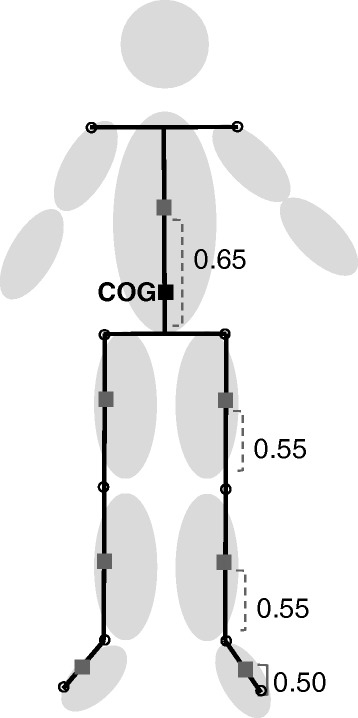
**COG (center of each segment).** The body was divided into seven segments (1, Trunk; 2 and 3, both the thighs; 4 and 5, both the lower thighs, and 6 and 7, both feet) defined by ten markers. The virtual value calculated with a software that estimated the equation of Ehara and Yamamoto [23] was considered as COG (Figure 3). Center of seven segments were calculated. COG was calculated as composition center of seven segments. *xg* = *m*_1_ *x*_1_ + *m*_2_ *x*_2r_ + *m*_3_ *x*_3r_ + *m*_4_ *x*_4r_ + *m*_2_ *x*_2l_ + *m*_3_ *x*_3l_ + *m*_4_ *x*_4l_; *yg* = *m*_1_ *y*_1_ + *m*_2_ *y*_2r_ + *m*_3_ *y*_3r_ + *m*_4_ *y*_4r_ + *m*_2_ *y*_2l_ + *m*_3_ *y*_3l_ + *m*_4_ *y*_4l_; *zg* = *m*_1_ *z*_1_ + *m*_2_ *z*_2r_ + *m*_3_ *z*_3r_ + *m*_4_ *z*_4r_ + *m*_2_ *z*_2l_ + *m*_3_ *z*_3l_ + *m*_4_ *z*_4l_. Mass ratios of each segment (*m*_1_: trunk 0.66, *m*_2_: thigh 1, *m*_3_: lower thigh 0.05, *m*_4_: foot 0.02) *x*_1_: center of trunk, *x*_2r_: center of right thigh, *x*_3r_: center of right lower thigh, *x*_4r_: center of right foot, *x*_2l_: center of left thigh, *x*_3l_: center of left lower thigh, *x*_4l_: center of left foot (Note: For information on how to calculate the center of seven segments, see Ehara and Yamamoto [23], pages 12 to 15).

**Figure 4:**
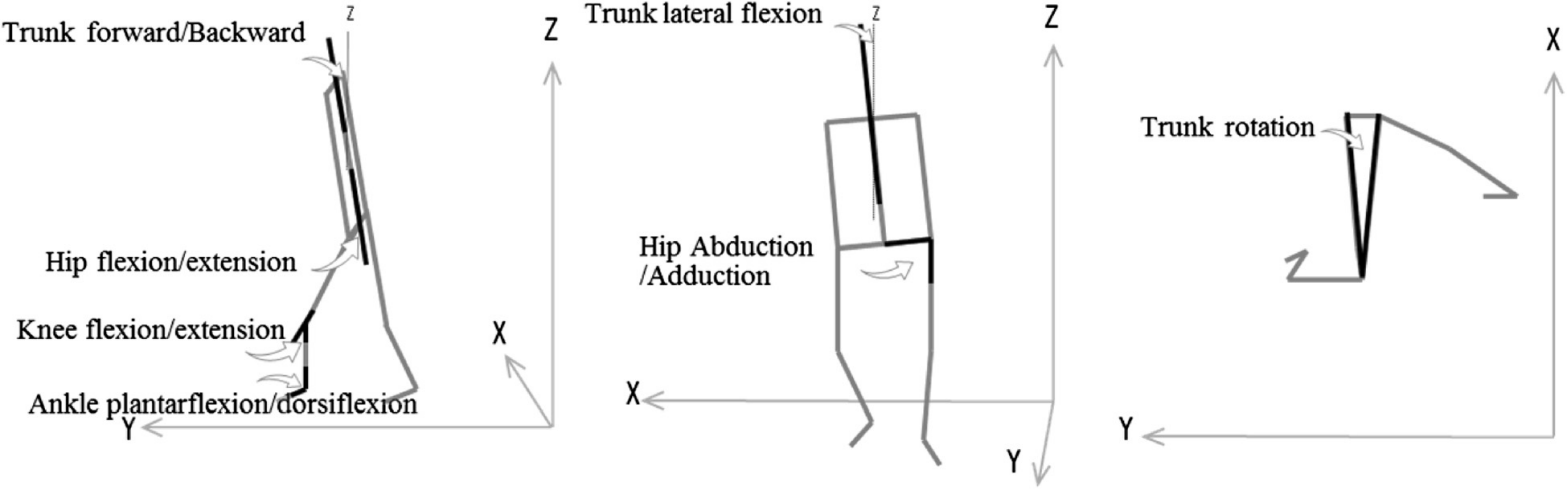
**Angle-related parameters.** Trunk flexion/extension, hip flexion/extension, knee flexion/extension, and ankle flexion/dorsiflexion were calculated in the sagittal plane. Trunk lateral flexion and hip abduction/adduction were calculated in the frontal plane; trunk rotation was calculated in the horizontal plane.

2-1) Height-related parameters (Figures 3 and 4)
  The distances between the ground and toe, heel, knee, COG, shoulder, and waist were recorded.
2-2) Angle-related parameters (Figure 4)
  The following movements were used to examine joint angles: hip flexion/extension, knee flexion/extension, ankle plantar flexion/dorsiflexion, trunk flexion/extension, trunk lateral flexion, hip abduction/adduction, and trunk rotation.

### Statistical analyses

To examine differences in the mean parameter values among different age groups and obstacle heights for each parameter at the initial contact instant, two-factor analysis of variance (ANOVA) with repeated measures on one factor was used. To compare means among age levels and obstacle heights for each parameter in which significant correlations with the height were observed, we performed an analysis of covariance (ANCOVA) in which height was used as a covariate. Multiple *post hoc* comparisons were performed using Tukey’s honestly significant differences test if a significant main effect or interaction was identified. A probability level of *P* < 0.05 was considered statistically significant. STATISTICA 5.1 (StatSoft Inc., Tulsa, USA) was used for all statistical analyses.

## Results

### Initial contact instant

Table 1 presents the mean and standard deviation (SD) for each parameter during the initial contact instant according to age and obstacle height. Trunk rotation showed a significant main effect for age. The multiple comparisons revealed that trunk rotation for all obstacle conditions was greater in the elderly women than in the young women.

**Table 1:** **Mean and standard deviation (SD) for each parameter during the initial contact instant**

	**Obstacle’s heights**	**The elderly**		**The young**			**ANOVA**		**Multiple comparison**	
**Parameters**		**Mean**	**SD**	**Mean**	**SD**		**F**	***P***	**Factor**	**Tukey’s HSD**
Hip flexion/extension [∠yz] (deg)	0 cm	23.7	7.7	27.6	7.5	F1	2.17	0.15		
	5 cm	21.0	8.3	25.0	9.3	F2	2.61	0.08		
	20 cm	19.7	6.7	25.5	9.9	F3	0.27	0.77		
Knee flexion/extension^a^ [∠yz] (deg)	0 cm	8.4	7.7	11.9	10.9	F1	1.32	0.26		
	5 cm	8.0	6.8	10.4	7.4	F2	0.56	0.57		
	20 cm	5.7	7.2	11.7	8.6	F3	0.85	0.43		
Ankle plantar flexion/dorsiflexion [∠yz] (deg); (90 − α)	0 cm	−16.7	9.4	−19.6	8.0	F1	0.32	0.58		
	5 cm	−20.0	5.6	−17.8	7.9	F2	0.97	0.39		
	20 cm	−23.1	7.1	−17.8	8.0	F3	2.93	0.06		
Hip abduction/adduction [∠yz] (deg); (−90 + α)	0 cm	−6.2	4.2	−9.8	3.3	F1	4.05	0.06		
	5 cm	−7.5	4.5	−9.4	4.5	F2	0.33	0.72		
	20 cm	−6.2	5.0	−9.0	4.5	F3	0.09	0.91		
Trunk lateral flexion^a^ [∠zx] (deg)	0 cm	1.9	1.2	1.7	0.8	F1	1.46	0.24		
	5 cm	2.3	1.4	1.1	0.4	F2	0.47	0.63		
	20 cm	2.9	1.8	1.2	0.6	F3	1.81	0.18		
Trunk forward/backward [∠yz] (deg)	0 cm	2.3	4.4	3.1	2.6	F1	0.61	0.44		
	5 cm	1.4	4.3	2.8	3.0	F2	2.62	0.08		
	20 cm	0.3	3.8	1.5	3.7	F3	0.09	0.91		
Trunk rotation^a^ [∠xy] (deg)	0 cm	11.5	6.2	22.8	4.1	F1	5.97*	0.02		
	5 cm	12.7	6.6	19.8	4.2	F2	0.17	0.85	Group	0, 5, 20 cm: E < Y
	20 cm	11.5	5.6	21.7	6.1	F3	1.02	0.37		
Center of gravity^a^ [z] (cm)	0 cm	76.0	3.0	84.0	3.5	F1	0.91	0.35		
	5 cm	76.3	3.2	85.6	4.3	F2	2.29	0.11		
	20 cm	76.0	2.6	85.0	4.5	F3	0.44	0.65		
Knee^a^ [z] (cm)	0 cm	35.1	3.0	38.6	3.8	F1	1.24	0.28		
	5 cm	34.8	2.7	38.7	4.5	F2	1.14	0.33		
	20 cm	33.6	1.9	38.7	4.3	F 3	1.58	0.22		
Ankle^a^ [z] (cm)	0 cm	4.6	0.9	6.1	1.0	F1	4.07	0.06		
	5 cm	4.7	0.8	5.6	1.8	F2	3.16	0.05		
	20 cm	4.2	0.5	5.4	1.7	F3	0.84	0.44		
Toe^a^ [z] (cm)	0 cm	3.9	1.2	4.8	1.9	F1	1.06	0.32		
	5 cm	3.1	1.2	4.5	2.9	F2	3.17*	0.05	Obst.	Elderly: 0 > 20
	20 cm	2.6	1.0	4.3	2.4	F 3	0.86	0.43		
Shoulder^a^ [z] (cm)	0 cm	111.0	4.4	125.3	5.0	F 1	0.00	0.99		
	5 cm	111.5	4.7	126.6	5.5	F 2	3 .0 6	0.0 6		
	20 cm	111.2	4.2	126.0	5.9	F3	0.64	0.53		
Waist^a^ [z] (cm)	0 cm	66.9	3.4	72.4	3.9	F1	1.35	0.26		
	5 cm	67.1	3.8	73.7	4.5	F2	2.40	0.10		
	20 cm	67.1	2.8	72.7	4.7	F3	1.47	0.24		

*P < 0.05.

F1: between age groups, F2: between obstacles, F3: interaction, E: the elderly, Y: the young.

^a^Because parameters were correlated with height, we performed ANCOVA, which utilized height as a covariate.

Toe height showed a significant main effect for obstacle height. The multiple comparisons revealed that toe height was higher for the 0-cm obstacle than for the 20-cm obstacle in the elderly women.

### Swing instant with the leading limb

Table 2 presents the mean and SD for each parameter during the swing instant with the leading limb according to age and obstacle height. Angles for hip flexion/extension, knee flexion/extension, ankle plantar flexion/dorsiflexion, hip adduction/abduction, and trunk lateral flexion showed a significant main effect for obstacle height, whereas trunk rotation showed a main effect for age. Multiple comparisons revealed that flexion at the hip and knee increased with an increasing obstacle height for both age groups. Ankle dorsiflexion and hip adduction angles for the elderly were greatest for the 0-cm and 5-cm obstacles than for the 20-cm obstacle.

**Table 2:** **Mean and SD for each parameter during the swing instant with the leading limb**

**Parameters**	**Obstacle’s heights**	**The elderly**		**The young**			**ANOVA**		**Multiple comparison**	
		**Mean**	**SD**	**Mean**	**SD**		**F**	***P***	**Factor**	**Tukey’s HSD**
Hip flexion/extension [∠yz] (deg)	0 cm	26.5	9.3	24.7	4.1	F1	1.02	0.32	Obst.	Elderly: 0 < 5 < 20
	5 cm	46.2	8.6	41.3	7.1	F2	187.96*	0.00		Young: 0 < 5 < 20
	20 cm	65.5	11.7	62.9	8.0	F3	0.33	0.72		
Knee flexion/extension [∠yz] (deg)	0 cm	30.0	8.8	32.3	4.2	F1	1.66	0.21	Obst.	Elderly: 0 < 5 < 20
	5 cm	52.8	10.6	62.7	14.0	F2	111.67*	0.00		Young: 0 < 5 < 20
	20 cm	84.2	16.0	85.4	16.9	F3	0.88	0.42		
Ankle plantar flexion/dorsiflexion [∠yz] (deg); (90-α)	0 cm	−16.9	6.7	−15.2	3.5	F1	0.22	0.65	Obst.	Elderly: 0, 5 < 20
	5 cm	−15.7	5.3	−11.4	6.6	F2	13.56*	0.00		
	20 cm	−6.1	9.0	−9.0	7.6	F3	2.38	0.10		
Hip abduction/adduction [∠yz] (deg); (−90 + α)	0 cm	−8.7	4.6	−9.3	4.3	F1	0.12	0.73	Obst.	Elderly: 0, 5 > 20
	5 cm	−12.1	8.4	−11.5	4.6	F2	13.04*	0.00		
	20 cm	−26.4	20.8	−22.5	13.0	F3	0.28	0.75		
Trunk lateral flexion [∠zx] (deg)	0 cm	1.7	1.2	1.2	0.8	F1	1.04	0 .3 2		
	5 cm	2.3	1.6	1.5	1.2	F2	6.54*	0.00	ns^a^	
	20 cm	2.5	1.4	2.4	1.2	F3	1.16	0.32		
Trunk forward/backward [∠yz] (deg)	0 cm	2.3	4.2	0.8	2.1	F1	0.72	0.40		
	5 cm	2.4	4.8	0.7	2.8	F 2	0 .1 9	0.83		
	20 cm	2.3	4.4	1.5	4.1	F3	0.28	0.76		
Trunk rotation [∠xy] (deg)	0 cm	8.2	4.7	11.9	2.6	F1	11.10*	0.00	Group	5 cm: E < Y
	5 cm	7.0	4.1	13.8	7.0	F2	0.05	0.95		
	20 cm	8.5	4.8	11.4	7.2	F3	0.93	0.40		
Center of gravity [z] (cm)	0 cm	78.2	2.9	88.0	4.4	F1	51.79*	0.00	Group	0, 5, 20 cm: E < Y
	5 cm	79.5	2.6	90.2	4.8	F2	98.41*	0.00	Obst.	Elderly: 0 < 5 < 20
	20 cm	81.7	2.4	93.3	4.8	F3	4.05*	0.02		Young: 0 < 5 < 20
Knee^b^ [z] (cm)	0 cm	39.6	3.0	43.1	3.3	F1	0.42	0.52		
	5 cm	48.8	3.3	51.8	3.2	F2	283.35*	0.00	Obst.	Elderly: 0 < 5 < 20
	20 cm	61.6	4.9	67.6	5.0	F3	1.32	0.28		Young: 0 < 5 < 20
Ankle [z] (cm)	0 cm	8.2	1.1	9.1	1.3	F1	14.64*	0.00	Group	5, 20 cm: E < Y
	5 cm	18.0	3.4	22.0	2.8	F2	520.71*	0.00		Elderly: 0 < 5 < 20
	20 cm	35.5	4.0	39.7	4.0	F3	2.12	0.13	Obst.	Young : 0 < 5 < 20
Toe [z] (cm)	0 cm	4.5	1.2	4.8	1.3	F1	3.10	0.09	Obst.	Elderly: 0 < 5 < 20
	5 cm	14.1	3.4	16.2	2.3	F2	464.01*	0.00		Young : 0 < 5 < 20
	20 cm	31.3	4.6	33.9	4.4	F3	0.89	0.42		
Shoulder^b^ [z] (cm)	0 cm	113.4	4.5	129.7	5.8	F1	3.00	0.10		
	5 cm	113.3	4.2	130.4	6.1	F2	3.06	0.06		
	20 cm	113.4	3.9	131.2	6.3	F3	2.84	0.07		
Waist^b^ [z] (cm)	0 cm	69.4	3.1	76.2	4.8	F1	0.58	0.46		
	5 cm	70.3	2.8	78.0	5.4	F2	39.18*	0.00	Obst.	Elderly: 0, 5 < 20
	20 cm	71.8	3.0	80.4	5.3	F3	2.76	0.07		Young : 0, 5 < 20

*P < 0.05.

F1: between age groups, F2: between obstacles, F3: interaction, E: the elderly, Y: the young.

Center of gravity was used in ANOVA because syntactic parallelism between age groups could not be assumed.

^a^The main effect on trunk lateral flexion is shown in Table 2, whereas no significant *post hoc* was observed in this parameter. Instead, we stated the effect sizes, which can refer to the raw difference between conditions in this parameter as shown in Table 2.

^b^Because parameters were correlated with height, we performed ANCOVA, which utilized height as a covariate.

Effect size: the elderly - 0 cm vs. 5 cm: 0.41, 0 cm vs. 20 cm: 0.55, 5 cm vs. 20 cm: 0.1; the young - 0 cm vs. 5 cm: 0.29, 0 cm vs. 20 cm: 1.23, 5 cm vs. 20 cm: 0.8.

COG and ankle heights showed a significant main effect for age; height-related parameters, except for the shoulder height, showed a significant main effect for obstacle height. COG height showed a significant interaction. Multiple comparisons revealed that COG heights for all obstacle conditions as well as ankle height for the 5-cm and 20-cm obstacles were greater in the young women than in the elderly women. The heights for COG, knee, ankle, and toe increased with an increasing obstacle height for both age groups, and waist height was greatest for the 20-cm obstacle than for the 0-cm and 5-cm obstacles.

### Swing instant with the trailing limb

Table 3 presents the mean and SD for each parameter during the swing instant with the trailing limb according to age and obstacle height. Angle-related parameters for hip and knee flexion/extension, ankle plantar flexion/dorsiflexion, trunk lateral flexion, and trunk forward/backward showed main effects for obstacle height; hip flexion/extension also showed a significant interaction. Multiple comparisons revealed that the hip flexion for the 5-cm and 20-cm obstacles were greater in the elderly women than in the young women. In addition, hip and knee flexion angles were roughly greater with an increasing obstacle height for both age groups. Ankle dorsiflexion, trunk lateral flexion, and trunk flexion angles in the elderly were greater for the 20-cm obstacle than for the 0-cm and 5-cm obstacles.

**Table 3:** **Mean and SD for each parameter during the swing instant with the trailing limb**

	**Obstacle’s heights**	**The elderly**		**The young**			**ANOVA**		**Multiple comparison**	
**Parameters**		**Mean**	**SD**	**Mean**	**SD**		**F**	***P***	**Factor**	**Tukey’s HSD**
Hip flexion/extension [∠yz] (deg)	0 cm	−3.1	10.2	−6.4	8.7	F1	7.16*	0.01	Group	5, 20 cm: E > Y
	5 cm	12.4	9.8	−1.6	9.4	F2	70.35*	0.00	Obst.	Elderly: 0 < 5 < 20
	20 cm	26.5	11.3	14.8	9.9	F3	3.43*	0.04		Young: 0, 5 < 20
Knee flexion/extension^a^ [∠yz] (deg)	0 cm	56.9	7.5	54.7	9.6	F1	2.44	0.13		
	5 cm	82.8	9.8	74.1	10.7	F2	190.46*	0.00	Obst.	Elderly: 0 < 5 < 20
	20 cm	115.3	7.7	103.3	10.8	F3	1.64	0.21		Young: 0 < 5 < 20
Ankle plantar flexion/dorsiflexion [∠yz] (deg); (90-α)	0 cm	−30.5	6.5	−36.9	7.6	F1	2.39	0.14		
	5 cm	−24.7	8.4	−30.4	9.7	F2	15.04*	0.00	Obst.	Elderly: 0, 5 < 20
	20 cm	−15.6	7.0	−26.8	13.3	F3	0.8 8	0.42		
Hip abduction/adduction [∠yz] (deg); (−90 + α)	0 cm	1.0	3.5	−0.8	3.5	F1	0.03	0.86		
	5 cm	−0.1	6.2	1.5	5.8	F2	1.59	0.21		
	20 cm	3.7	6.6	1.8	9.5	F3	1.26	0.29		
Trunk lateral flexion^a^ [∠zx] (deg)	0 cm	1.8	1.4	1.1	0.8	F1	0.38	0.55		
	5 cm	2.5	1.8	1.6	1.3	F2	8.96*	0.00	Obst.	Elderly: 0, 5 < 20
	20 cm	4.3	2.3	2.2	1.3	F3	1.68	0.20		
Trunk forward/backward^a^ [∠yz] (deg)	0 cm	−1.8	4.3	−2.9	2.0	F1	0.61	0.44		
	5 cm	0.7	4.6	-3.1	2.6	F2	22.99*	0.00	Obst.	Elderly: 0, 5 < 20
	20 cm	5.0	4.8	0.3	3 .2	F 3	2 .9 0	0.07		
Trunk rotation [∠xy] (deg)	0 cm	8.4	4.5	8.3	5.3	F1	1.15	0.30		
	5 cm	5.5	5.1	10.6	6.6	F2	1.67	0.20		
	20 cm	6.1	5.9	5.1	4.1	F3	1.96	0.15		
Center of gravity^a^ [z] (cm)	0 cm	77.1	2.6	86.1	3.8	F1	0.03	0.87	Obst.	Elderly: 0, 5 < 20
	5 cm	77.6	2.7	86.9	4.1	F2	47.06*	0.00		Young: 0, 5 < 20
	20 cm	80.0	2.6	89.1	4.7	F3	0.10	0.90		
Knee [z] (cm)	0 cm	35.1	2.4	37.1	2.6	F1	3.09	0.09	Obst.	Elderly: 0 < 5 < 20
	5 cm	37.5	2.5	39.4	3.1	F2	86.39*	0.00		Young: 0, 5 < 20
	20 cm	43.0	3.1	44.9	4.4	F3	0.01	0.99		
Ankle [z] (cm)	0 cm	19.6	1.3	20.9	2.7	F1	0.69	0.41	Obst.	Elderly: 0 < 5 < 20
	5 cm	28.2	3.0	29.8	3.3	F2	342.62*	0.00		Young: 0 < 5 < 20
	20 cm	44.7	3.9	44.4	5.6	F3	0.56	0.57		
Toe [z] (cm)	0 cm	9.4	1.1	9.1	1.5	F1	0.21	0.65	O b st.	Elderly: 0 < 5 < 20
	5 cm	18.0	3.1	18.8	3.7	F2	319.63*	0.00		Young: 0 < 5 < 20
	20 cm	35.9	4.3	34.2	6.1	F3	0.74	0.48		
Shoulde r^a^ [z] (cm)	0 cm	112.0	3.9	127.5	4.9	F1	3.36	0.08		
	5 cm	111.9	4.1	127.7	5.2	F2	10.04*	0.00	Obst.	Young: 0 < 20
	20 cm	113.0	4.1	129.2	6.0	F3	0.4 4	0.64		
Waist^a^ [z] (cm)	0 cm	67.8	3.2	73.5	4.4	F1	0.27	0.61	Obst.	Elderly: 0, 5 < 20
	5 cm	67.8	3.2	74.1	4.8	F2	18.31*	0.00		Young: 0 < 20
	20 cm	69.9	3.3	75.5	5.2	F3	0.44	0.64		

*P < 0.05.

F1: between age groups, F2: between obstacles, F3: interaction, E: the elderly, Y: the young.

^a^Because parameters were correlated with height, we performed ANCOVA, which utilized height as a covariate.

Hip flexion/extension was used ANOVA because syntactic parallelism between age groups could not be assumed.

All height-related parameters showed a significant main effect for obstacle height. Multiple comparisons showed that COG, knee, ankle, toe, and waist heights were roughly higher in the order of 20-cm, 5-cm, and 0-cm obstacles for both age groups, and shoulder height was higher for the 20-cm obstacle than for the 0-cm in the young women.

## Discussion

A decline in obstacle-crossing performance with age may be implied by the higher incidence of tripping and stumbling in older adults [18]. We performed three-dimensional motion analysis after dividing the movement instants into initial contact and swing instants.

### Initial contact instant

The elderly women showed a preparation posture with a low toe height before stepping over the obstacles, which is characteristic of stepping over obstacles in the initial contact instant. Although the toe height in the young women did not change with obstacle heights, the elderly women showed a lower toe height that decreased with obstacle height. It is assumed that the elderly women decreased their walking speed by decreasing their toe height before stepping over the obstacle. This movement pattern in the elderly women may have resulted from a fear of crossing obstructions; alternatively, it could be a strategy for stepping over the obstacles with care. Galna et al. [19] reported that adoption of a conservative strategy with age may help in explaining why older individuals showed such few obstacle contacts during locomotion.

On the other hand, the young women exhibited greater trunk rotation. This pattern may depend on a difference in stride. Because of elderly individuals’ decreased strength and balance, they increase their postural stability by decreasing their stride distance. Therefore, their trunk rotation may be smaller. Chou et al. [14], who examined the effect of toe-obstacle distance in individuals stepping over obstacles, reported that as the toe-obstacle distance decreased, the maximum plantar flexion moment at the ankle significantly decreased just after heel contact. This direct relationship was likely due to a decrease in crossing speed in preparation for crossing the obstacle, which can be expected to have led to a decrease in the vertical component of the ground reaction force tending to plantar flex the ankle.

### Swing instant

At the swing instant of the leading limb, the height of the waist and COG was higher with higher obstacles for both age groups; however, the shoulder height did not change. It is inferred that both elderly women and young women tilted their upper bodies to the side when stepping over obstacles. In contrast, among the elderly women, ankle dorsiflexion and hip adduction were greatest when they stepped over the 20-cm obstacle. Because of decreased lower-limb strength, the elderly women stepped over the obstacle with a dorsiflexed ankle and an adducted hip [20]. This movement pattern may be their ideal movement strategy.

At the swing instant with the trailing limb, the elderly women showed large ankle dorsiflexion, knee and hip flexion, and trunk lateral flexion when they stepped over the 20-cm obstacle. The elderly were able to confirm the obstacle height visually during the swing phase with the leading limb; however, they could not confirm the obstacle height during the swing phase with the trailing limb. Therefore, they performed a swing with the trailing limb on the basis of visual information obtained during the swing phase with the leading limb. Increased ankle dorsiflexion with the trailing limb may be performed without foot-obstacle contact. In this case, impairments in joint mobility at the hip may diminish proprioception of lower extremity. Chen et al. [21] reported that a participant group with joint disorders crossed the obstacle with a higher trailing toe clearance. This strategy may be helpful for decreasing the probability of the foot hitting the obstacle and thus decreasing the risk of tripping when the swing limb and obstacle are not in the subject’s visual field [21]. In addition, the elderly maintained a position of anteversion when they stepped over obstacles. We infer that the elderly moved the trailing limb with a position of anteversion because they cannot lift the foot high above the ground.

Kim [6] described gait characteristics of the elderly as large lateral lean, small up-and-down swaying movements, a small hip angle, and smaller ankle plantar flexion angle during contact with the ground. In addition, the movements of the elderly women as they stepped over the obstacles in this study were similar to those described in previous studies. Although swaying-up-and-down movements were not observed in the present study, lateral lean was observed in all phases. Because of their decreased balance, the elderly maintained their postural stability primarily by increasing their base of support (large step width). Therefore, their lateral lean may have increased.

An important finding in the present study is the elderly women’s tendency of bending their trunk forward and showing lateral tilting of their upper body during the trailing limb swing instant. This pattern may be a strategy that elderly individuals with poor lower-limb strength adopt when there is a need to step over high obstacles. However, no thanks to the anterior and lateral inclination of their posture, the elderly may have a high risk of falling when tripping or losing balance during a one-leg stance. Lu et al. [10] reported that the existing knowledge of kinematic control during obstacle crossing is based mostly on young subjects and may serve as a baseline for studies with elderly individuals. Such studies may improve the understanding of the mechanisms underlying falls in the elderly women and subsequent prevention. To improve stepping movements while navigating obstacles, it may be necessary for elderly individuals to decrease their fear of obstacles [22], and this mentality may be achieved with regular training. In addition, it is important to strengthen the iliopsoas and quadriceps muscles that generally lift the foot and maintain trunk stability while stepping over obstacles.

In sum, the elderly women prepared for stepping over the obstacle carefully at the initial contact instant. In the swing instant, they showed greater ankle dorsiflexion and hip adduction angles with the leading limb when stepping over the 20-cm obstacle. Compared with young women, the elderly women moved the trailing limb with increased ankle dorsiflexion, knee flexion, hip flexion, and foot inversion to ensure that they did not touch the obstacle as they stepped over it. In the local community setting, exercise programs combined with stepping-over-obstacle activities need to be held for elderly women so that they would become more able to recognize obstacle height precisely as well as to train thigh muscles for lower limb lifting. In addition, the elderly need to understand the characteristics of their movements while stepping over obstacles as well as try to change their movements more consciously if there are risks of tripping or wobbling.

### Limitations of this study

The present study used 20-cm-high obstacles, similar to stair heights encountered in daily living, and 5-cm-high ones. These obstructions simulate day-to-day conditions wherein the elderly are likely to trip easily. In future studies, we aim to use various obstacle conditions that are based on the height and length of the participants’ lower limbs. In the present study, we examined the initial contact and swing instants for the leading and trailing limbs on the basis of seven joint angles and the distances between the ground and six markers. It is necessary to study the characteristics of stepping over obstacles in the elderly from various viewpoints that include joint mobility, proprioceptive sense, and lower-limb strength. In addition, it is necessary to evaluate the movements of the elderly while stepping over obstacles because of these individuals’ high risk of tripping or falling.

## Conclusions

In conclusion, in the initial contact instant, the elderly women prepared for stepping over the 20-cm obstacle with a decreased toe height. Trunk rotation was greater in the young women than in the elderly women.

In the swing instant, the elderly women showed greater ankle dorsiflexion and hip adduction in the leading lower limb when stepping over the 20-cm obstacle. Compared with the young women, the trailing lower limb in the elderly women had increased ankle dorsiflexion, knee flexion, hip flexion, and foot inversion to ensure that they did not touch the obstacle as they stepped over it. This movement pattern is considered to be characteristic of elderly individuals who are unable to lift their lower limb straight up from the ground because of decreased lower limb strength.
